# Primary Spinal Melanoma With Intra- and Extradural Extensions: A Rare Case

**DOI:** 10.7759/cureus.12855

**Published:** 2021-01-22

**Authors:** Noman Saleem, Rabia Saleem, Hannan Asghar, Musab Zubair, Umar Farooque

**Affiliations:** 1 Forensic Medicine, Sahiwal Medical College, Sahiwal, PAK; 2 Neurosurgery, Punjab Institute of Neurosciences, Lahore, PAK; 3 Internal Medicine, Orange Park Medical Center, Orange Park, USA; 4 Medicine, DHQ Teaching Hospital, Sahiwal, PAK; 5 Neurology, Dow University of Health Sciences, Karachi, PAK

**Keywords:** primary spinal melanoma, space-occupying lesion, extradural, malignant, laminectomy

## Abstract

Primary spinal melanoma (PSM) is a rare primary central nervous system melanoma with limited literature. A 30-year-old male presented with one year of progressive bilateral leg weakness and back pain. Physical examination revealed slightly decreased power and deep tendon reflexes of the lower extremities, decreased sensation at the level of T10, and normal anal sphincter and plantar reflexes. Magnetic resonance imaging (MRI) scan of the thoracolumbar spine revealed a hypointense lesion on T2-weighted and a hyperintense lesion on T1-weighted imaging at the level of T10 with mild extensions. The lesion was causing a mass effect on the spinal cord. The patient underwent laminectomy and near-total excision which showed a black, firm-to-hard, scarcely vascular extradural tumor extending from T10 to T11 that adhered to nerve roots. Histopathological examination and immunostaining with S-100 and Melan-A stains confirmed the diagnosis of malignant melanoma. Other imaging studies like brain computed tomography (CT) and positron emission tomography/computed tomography (PET/CT) scans, and chest X-ray were normal. On follow-up, the patient reported improvement in the power of his lower limbs with intact sensory function and sphincters. The first radiotherapy session was scheduled for six weeks postoperatively. There was no recurrence at a two-year follow-up. The possibility of a melanocytic tumor should be considered for a spinal lesion with paramagnetic properties as early surgical intervention is important for diagnosis and improved survival.

## Introduction

Malignant melanoma is a cancer of melanocytes that can arise from any melanin-containing cell. Primary central nervous system (CNS) melanoma, however, is rare. It comprises only 1% of melanomas which include blue nevus, meningeal melanocytoma, melanotic schwannoma, and malignant melanoma [1]. Primary spinal melanoma (PSM) is an even rarer entity. Hirschberg first reported PSM in 1906 and Hayward first classified primary CNS melanoma, differentiating it from metastatic melanoma [2, 3].

Due to the rarity of PSM, the literature on the epidemiology, natural history, diagnostic/therapeutic approach, and prognosis is not vast. To our knowledge, we are reporting the first case of extradural PSM with intradural extension from Pakistan.

## Case presentation

A 30-year-old male patient presented with a complaint of bilateral leg weakness for one year. The weakness started gradually in the right leg followed by the involvement of the left leg. It was associated with progressive middle back pain that was aching in nature, 4/10 in intensity, and worse at night. He denied any stiffness, numbness, tingling, urinary/bowel incontinence, skin changes, or fever/chills. Personal, family, and social histories were non-contributory.

Physical examination of bilateral lower and upper extremities revealed power grades of 3/5 and 5/5, respectively. The patient had slightly diminished deep tendon reflexes (DTRs) in the ankles and knees bilaterally. Neurological examination of the back revealed diminished sensations at the level of T10. Plantar and anal sphincter reflexes were intact. Skin and eye examinations were normal. Due to chronic leg weakness and nocturnal back pain, the patient was admitted to the hospital for imaging studies.

Diagnosis

**Figure 1:**
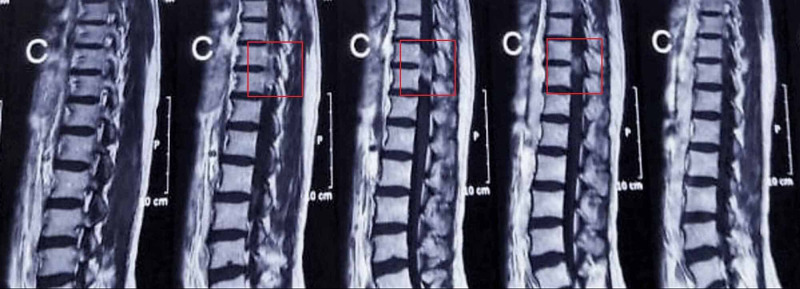
Magnetic resonance imaging scan (sagittal plane) of the thoracolumbar spine. The red boxes are showing the hyperintense lesion at level of T10-T11 on T1-weighted MRI.

Magnetic resonance imaging (MRI) scan of the thoracolumbar spine revealed a 1.5 x 1.2 cm hypointense lesion on T2-weighted (T2-W) imaging and a hyperintense lesion on T1-weighted (T1-W) imaging at the level of T10-T11 with mild intradural and extradural extensions (Figure 1). The lesion was causing a mild mass effect on the spinal cord. Given the scarcity of primary spinal space-occupying lesions, we considered ependymoma, meningioma, melanoma, epidermoid tumor, and infection as our differential diagnoses.

Treatment

**Figure 2:**
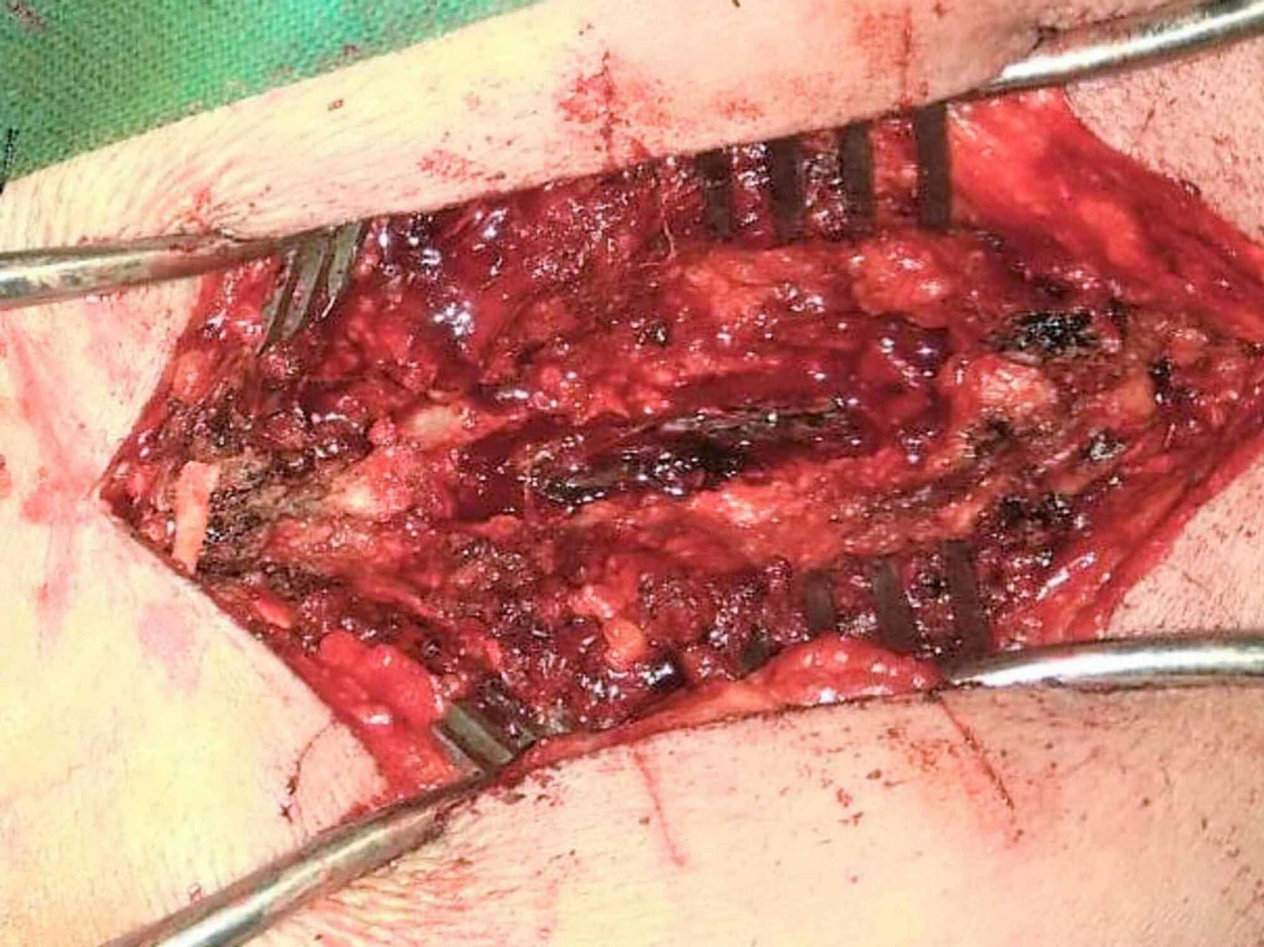
Intraoperative findings.

The lesion was causing a mild mass effect on the spinal cord. Due to these the patient underwent laminectomy and near-total excision of the mass. Partial laminectomy of T9 and T12 and complete laminectomy of T10 and T11 were performed. The neurosurgeon identified a black, firm-to-hard, scarcely vascular extradural tumor with intradural extension that was extending from T10 to T11. The intraoperative findings are shown in Figure 2. The tumor was adherent to the nerve root, more on the right side.

Pathological findings

**Figure 3:**
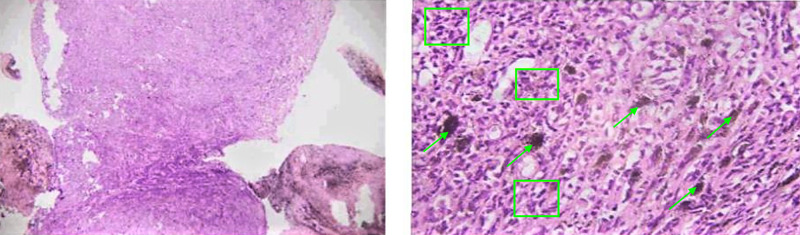
Microscopic findings on histological examination of the tumor. As shown in microscopic examination of mass, areas of excessive pigmentation (green arrows), nuclear pleomorphism and hyperchromasia (green boxes) are seen.

Histopathological examination of the excised tissue revealed an extensively pigmented malignant neoplasm arranged in bundles, sheets, and fascicles. Tumor cells were spindle-oval shaped, having hyperchromatic pleomorphic nuclei with high mitotic activity (Figure 3). These findings were suggestive of malignant melanoma. On immunohistochemistry, S-100 and Melan-A stains were positive which confirmed the diagnosis. Chest X-ray, brain computed tomography (CT) scan, and positron emission tomography/computed tomography (PET/CT) scans were normal and they were performed preoperatively.

Follow-up and outcomes

On follow-up after three weeks, the patient reported improvement in the power of his lower limbs and had started to walk with support. Neurological examination revealed a power grade of 4/5 bilaterally in the lower extremities. Sensory function and sphincters were intact. The first radiotherapy session was scheduled for six weeks post-operatively. At a two-year follow-up, the patient has not had a recurrence and it is confirmed by repeating the MRI of spinal cord.

## Discussion

We have presented a case of extradural primary spinal melanoma of the thoracic spinal region with intradural extensions. Primary pigmented lesions, including blue nevus, meningeal melanocytoma, melanotic schwannoma, and primary malignant melanoma of the CNS, are rare [4-7]. Primary spinal melanoma of the CNS accounts for only 1% of these CNS lesions and PSM comprises an even smaller percentage [1]. It can arise from within or outside the spinal cord. Primary leptomeningeal, nerve root, extradural, and intradural (including extramedullary and intramedullary) lesions have been reported [4-11]. Rarely, nerve root tumors can expand into intervertebral foramina and even extend beyond them [11]. The clinical picture of PSM is non-specific and similar to that of neurofibroma, meningioma, and ependymoma which often leads to preoperative misdiagnosis [9-11].

Malignant melanoma arises from melanin-containing cells in any organ containing melanocytes. The cellular origin of PSM is unclear. Multiple hypotheses, including dysembryogenetic and mesodermal, explaining the cellular origin have been proposed and discussed in the available literature [6]. Primary spinal melanoma most commonly affects the middle and lower thoracic spine [1, 3-7, 12]; the most prevalent PSM variant in the thoracic region is intramedullary PSM [1, 8]. On the other hand, extramedullary PSM has shown a slight propensity towards the cervical region [9].

Primary spinal melanoma mostly occurs in middle-aged individuals, affecting both genders equally [4-6, 12]. It commonly presents with backache and compressive spinal symptoms that are progressive and often asymmetric, such as the weakness of extremities seen in our patient [12]. According to Hayward, a CNS melanoma is considered primary if it is absent outside the CNS and at other sites within the CNS except for the primary tumor, and the diagnosis of melanoma is made with histopathology [3]. Our case satisfied Hayward's criteria. It is important to note that in most instances, CNS melanoma is metastatic, and a meticulous workup is essential to rule out visceral involvement [6].

Magnetic resonance imaging (MRI) is the best imaging modality to establish a preliminary diagnosis of spinal tumors. Malignant CNS melanoma characteristically appears hyperintense on T1-W and hypointense on T2-W MRI. In contrast studies, mild homogenous enhancement is seen [1, 13]. Woodruff et al. reported that tumor hemorrhage influenced paramagnetic appearance more significantly than melanin [13]. The characteristic MRI appearance helps to differentiate PSM from other spinal tumors such as meningioma or schwannoma. However, it cannot distinguish primary malignant melanomas from other pigmented CNS lesions such as leptomeningeal melanoma, melanocytoma, and metastatic malignant melanoma [14]. In such cases, an accurate diagnosis is only possible by histopathological examination.

There is no standardized treatment for PSM, but the therapeutic approach differs greatly from that of metastatic melanoma. Surgical resection of the tumor is the treatment of choice. The goal of surgery is therapeutic as well as diagnostic. Adjuvant radiotherapy may help in limiting tumor dissemination [12]. It is not clear whether adjuvant therapy plays an important role in increasing overall survival time [10-11, 15].

Due to limited data, the prognostic parameters of PSM and its overall survival are not certain. A retrospective study reported that tumor dissemination and chemo/radiotherapy without surgical resection were associated with the worst prognosis in PSM cases [12]. In patients with PSM, 12-month and 72-month survival rates were 89.6% and 39.6%, respectively [12]. Primary spinal melanoma has shown late recurrence and its survival time can vary drastically from three months to 21 years [7, 16].

In literature, PSM has been reported as an indolent tumor as compared to metastatic melanoma. The average survival in PSM patients after surgical resection was six years and seven months [4]. However, patients with metastatic malignant melanomas have significantly shorter average survival rates [17]. It shows that primary CNS melanoma has a higher life expectancy and an accurate diagnosis with early surgical resection can reduce the physical and psychological debilitation of patients.

## Conclusions

We must be cautious with the diagnosis of PSM. The possibility of melanocytic tumor should be considered for a spinal lesion with paramagnetic properties. Our case emphasizes that early surgical intervention to obtain a histological diagnosis is important and improves survival in patients with PSM.
